# Designing perfect linear polarization converters using perfect electric and magnetic conducting surfaces

**DOI:** 10.1038/srep38925

**Published:** 2016-12-13

**Authors:** Gaochao Zhou, Xudong Tao, Ze Shen, Guanghao Zhu, Biaobing Jin, Lin Kang, Weiwei Xu, Jian Chen, Peiheng Wu

**Affiliations:** 1 School of Electronic Science and Engineering, Nanjing University, Nanjing, 210023, China

## Abstract

We propose a kind of general framework for the design of a perfect linear polarization converter that works in the transmission mode. Using an intuitive picture that is based on the method of bi-directional polarization mode decomposition, it is shown that when the device under consideration simultaneously possesses two complementary symmetry planes, with one being equivalent to a perfect electric conducting surface and the other being equivalent to a perfect magnetic conducting surface, linear polarization conversion can occur with an efficiency of 100% in the absence of absorptive losses. The proposed framework is validated by two design examples that operate near 10 GHz, where the numerical, experimental and analytic results are in good agreements.

Polarization is an important characteristic of electromagnetic (EM) waves. In many related applications such as life science microscopy, fiber-optic communication and radar remote sensing, it is often required that the polarization states of the light must be controlled at will. To this end, conventional approaches are well-developed by taking advantage of birefringent crystals, Faraday rotations or Brewster angle effects^1^. These approaches, although prevail in modern laboratories, are unfortunately bulky and heavy, and thus being not suitable for miniaturized integrations. Such a problem can be mitigated with the help of advanced designs based on metasurfaces, as witnessed in the past decade^2^,^3^,^4^,^5^,^6^,^7^,^8^,^9^,^10^,^11^. The building elements of the metasurfaces are the so-called unit cells, of which the dimensions are much smaller comparing to the working wavelengths. By precisely tailoring the electromagnetic responses of the unit cells, various functional devices such as polarization converters^12^,^13^,^14^,^15^,^16^,^17^,^18^,^19^,^20^,^21^,^22^, flat lenses^8^,^11^,^23^ and phase gradient mirrors^24^,^25^,^26^ have been realized with subwavelength scale sizes, thereby opening a new route to manipulating light properties in arbitrary manners^9^,^21^,^27^,^28^,^29^,^30^.

Among various polarization manipulating devices, linear polarization converter is perhaps the most widely used one. A linear polarization converter rotates the polarization direction of a linearly polarized electromagnetic wave by 90 degree, making it perpendicular to the original one. Owing to its indispensable importance in many practical situations, linear polarization converter has attracted considerable amount of research efforts in recent years. Stimulated by these efforts, a variety of metasurface related linear polarization converters have been developed, such as those based on the chirality effect^12^,^13^,^31^,^32^,^33^, polarization dependent resonance effect^34^,^35^,^36^,^37^,^38^ and stacked grating effect^14^,^39^,^40^, just to name a few. These innovative polarization converting devices are expected to lead to significant technical advances in areas such as microwave and terahertz electronics^12^,^40^, where unlike the case of optical electronics, advantages of broad bandwidth and compact sizes are difficult to be offered by their traditional counterparts.

In this article, we present a design framework for perfect linear polarization converters. In the Result section, we first provide an intuitively explanation of the underlying principle. It is shown that perfect linear polarization conversion can be achieved if the device under consideration simultaneously possesses two symmetric mirror planes, with one being equivalent to a perfect electric conducting surface and the other being equivalent to a perfect magnetic conducting surface, depending on the way that the device is virtually excited. Following that picture, we next utilize metasurfaces to physically construct the required dual perfect conducting surfaces. Two different realization examples operating near 10 GHz are given. Numerical, experimental and analytic works carried out on these two realization cases confirm the predicted perfect linear polarization conversions, thereby provide a solid validation of the proposed design framework for perfect linear polarization converters.

## Result

### Design framework

**Figure 1:**
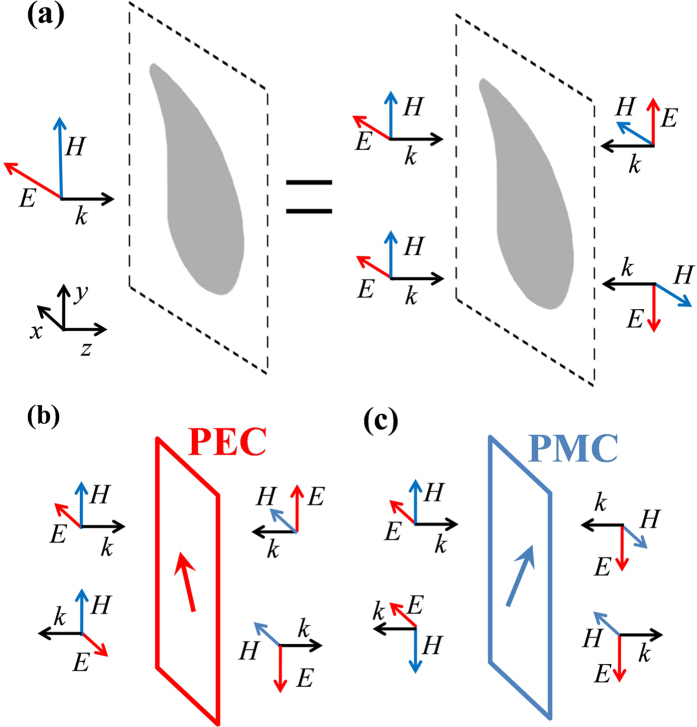
Principle of the design framework. (**a**) An x-polarized wave incident from the left side of a metasurface can be decomposed as a superposition of two virtual bi-directional excitations. (**b**) Illustration of the first type of virtual bi-directional excitation, which is referred as the E excitation. (**c**) Illustration of the second type of virtual bi-directional excitation, which is referred as the H excitation.

To illustrate the working principle of the proposed design framework, consider a lossless metasurface under illumination of an x-polarized electromagnetic wave incident from the left side. As shown by Fig. 1(a), such a one-directional excitation can be decomposed as a superposition of two virtual bi-directional excitations, where the two imaginary sources at the right side of the metasurface are polarized along the positive and negative directions of the y axis respectively. For clarity, the former virtual bi-directional excitation is referred as the E excitation while the latter virtual bi-directional excitation is referred as the H excitation. Without much effort, it is ready to see that at the plane of the metasurface, the electric field (and also the associated magnetic field) of the E excitation is polarized along the 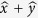 direction while the magnetic field (and also the associated electric field) of the H excitation is polarized along the 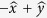 direction. Assuming that the metasurface has an electric type resonance if the driving electric field is polarized along the 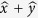 direction, for E excitation it will then behave as a perfect electric conducting surface at resonances. As a consequence, the two virtual waves incident from the left and right sides will be totally reflected, but with a π phase shift being imposed on the reflected electric field. Such a situation is illustrated in Fig. 1(b). On the other hand, assuming that the metasurface has a magnetic type resonance if the driving magnetic field is polarized along the 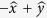 direction, for H excitation it will then behave as a perfect magnetic conducting surface. As illustrated in Fig. 1(c), a π phase shift will be imposed on the magnetic field of the two reflected virtual waves. When these two scenarios have a degenerate operating frequency, by taking a superposition of the reflected electromagnetic waves at that frequency, it follows that the overall reflected field is completely cancelled out at the left side of the metasurface and the electromagnetic field is 100% transmitted with the electric component being polarized along the y direction. In other words, perfect linear polarization conversion can be realized in this manner.

### Device realizations

The theoretical framework outlined above suggests that in order to achieve perfect linear polarization conversion, the metasurface needs to possess two degenerate and complementary resonances, with one being electric and the other being magnetic. To validate the framework and elaborate more on its principle, we design two types of perfect linear polarization converters that operate near 10 GHz by using different methods to realize the required dual resonances.

### Design case I: a narrow band linear polarization converter

**Figure 2:**
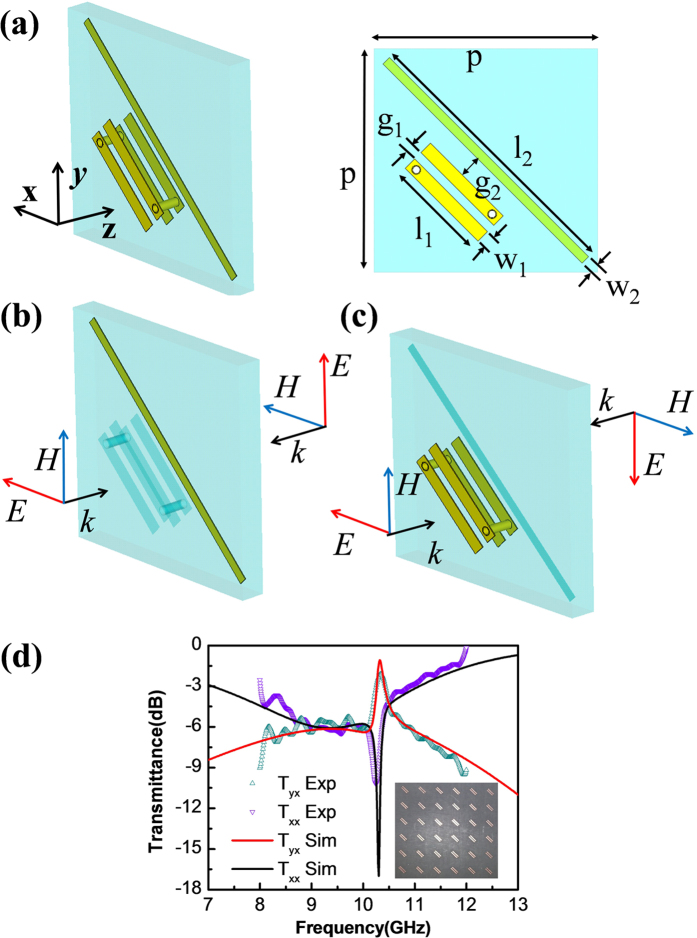
Design case I. (**a**) Schematics of the proposed device. (**b**) The proposed device under E excitation, where the cut-wire is the only activated element. (**c**) The proposed device under H excitation, where the double-U is the only activated element. (**d**) Numerically simulated and experimentally measured power transmittances for the converted and unconverted polarization components. The inset shows the photograph of the fabricated device. Note that the cut-wire and the double-U are degenerate in term of resonant frequencies.

Figure 2(a) sketches the unit cell of the linear polarization converter of the first case, which consists of a double-U structure and a cut-wire embedded in a thin substrate layer. Note that the double-U structure and the cut-wire are parallel to each other and are geometrically oriented along the 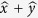 direction. Shown by Fig. 2(b), under case of E excitation, because the overall driving electric and magnetic fields are along the 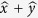 direction, the double-U structure is deactivated, leaving the cut-wire as the only active component. When properly tuned in resonance, the cut-wire behaves as a perfect electric conducting surface. On the other hand, as shown by Fig. 2(c), under case of H excitation, because the overall driving electric and magnetic fields are along the 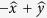 direction, the cut-wire is deactivated, leaving the double-U structure as the only active component. In this case, if tuned properly, the double-U structure shall act as a perfect magnetic conducting surface. According to the proposed general design framework, when the double-U structure and the cut-wire have degenerate resonant frequencies, perfect linear polarization then can be achieved. It should be noted that the magnetic resonance of the double-U structure originates from the induced magnetic flux flowing across the section of the double-U structure and in principle can also be provided with single-U structures^41^. Here we choose to use the double-U structure because it enables a more compact device size when comparing to the single-U case.

We first use finite element method based numerical simulations to confirm the above theoretical prediction. Illustrated by the right part of Fig. 2(a), the parameters of the simulated unit cell are l_1_ = 4.65 mm, w_1_ = 0.6 mm, g_1_ = 0.4 mm, l_2_ = 12.4 mm, w_2_ = 0.4 mm, g_2_ = 1 mm and p = 10 mm respectively. Note that these parameters are determined in such a way that the double-U structure and the cut-wire have degenerate resonant frequencies near 10 GHz. In our simulations, the double-U structure and cut-wire are assumed to be made of copper, whose conductivity is 5.8 × 10^7^ S/m. The substrate is assumed to be made of a 1.0 mm thick F4BK slab, of which the permittivity is 2.25 and the loss tangent is 0.0009. The solid lines in Fig. 2(d) plot the numerically obtained power transmittances for the converted and unconverted polarization components (denoted by T_xy_ and T_xx_ respectively therein). It can be seen that due to the deliberately tuned resonance degeneracy, there exists a narrow peak standing upon a broad peak in the transmittance curve for the converted component. The narrow peak corresponds to the resonance of the double-U structure while the broad peak corresponds to the resonance of the cut-wire. A constructive interference of these two peaks therefore leads to an enhanced polarization conversion efficiency at ~10 GHz. We remark that the peak value of the polarization conversion efficiency is ~80% instead of 100%. Such a discrepancy is attributed to be primarily caused by the absorption of the unit cell. Methods for reducing the conversion loss will be presented in the Discussion section. Moreover, in order to provide further validations, experimental works have also been carried out. The inset of Fig. 2(d) shows the fabricated device, which has a small thickness of 1 mm and a lateral size of 15 cm. The experimental data taken from the fabricated sample for the transmittances of the polarization converted and unconverted components are plotted by the symbols in Fig. 2(d). A good agreement between the numerical and experimental results can be identified.

The perfect linear polarization conversion process can be analytically treated by adopting the wave scattering picture. In such a picture, the overall field is regarded as a superposition of the incident wave and the scattered wave. Relating to design case I, the incident wave is from the realistic excitation source placed at the left side of the metasurface and reads as


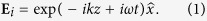


The scattered wave is from the electrical currents that flow on the metasurface and thus should be calculated in accordance to the way that the metasurface is excited. For the case of E excitation, only the cut-wire is activated. Since the electrical current flowing on the cut-wire is oscillating along the 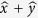 direction, the scattered field, or equivalently the field radiated by the cut-wire current, would also be polarized along the 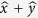 direction. Taking such a consideration into account, the scattered field then can be written as


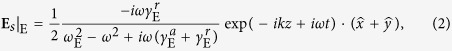


where the first factor of 1/2 describes the amplitude of the scattered field, and the second factor describes the electric resonance experienced by the current excited on the cut-wire, of which the resonant frequency is *ω*_E_ and the absorptive and radiative damping rates are 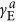 and 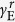 respectively^35^. We emphasize that the coefficient of 1/2 is determined from the fact that when the cut-wire is in resonance, it should behave as a perfect electric conducting surface in the absence of absorptive losses. As a result, the electric field radiated by the cut-wire and that by the two virtual sources of the E excitation should be of the same amplitude but out of phase at the cut-wire plane position. Following a similar treatment, for the case of H excitation, the scattered field, or equivalently the field radiated by the electrical current flowing on the double-U structure, reads as


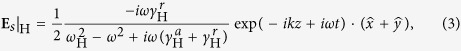


where the second factor describes a magnetic resonance experienced by the current excited on the double-U structure, of which the resonant frequency is *ω*_H_ and the absorptive and radiative damping rates are 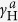 and 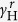 respectively. The overall field at the right side of the metasurface can be obtained by taking a summation over the field components given by Eqs (1, 2, 3), leading to the following expressions for the amplitude transmittances of the converted and unconverted polarization components


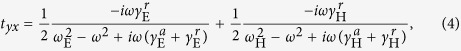



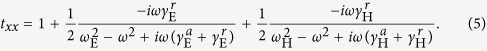


**Figure 3:**
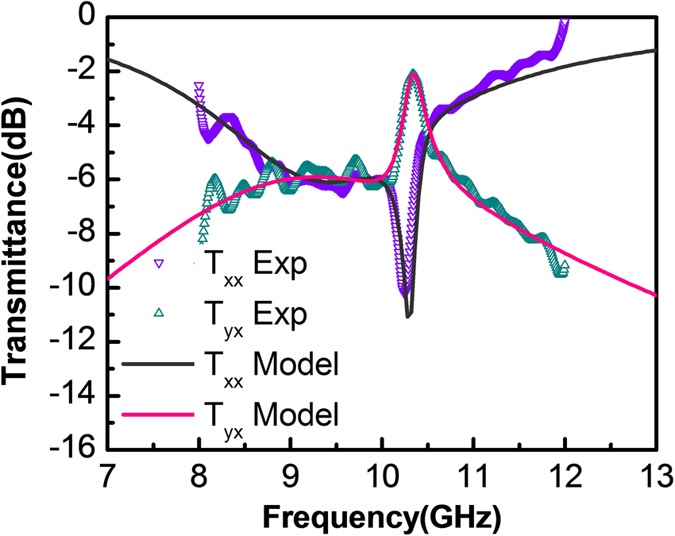
Analytically calculated and experimentally measured power transmittances of the converted and unconverted polarization components for design case I.

The analytic results obtained from Eqs (4 and 5) are plotted in Fig. 3 in together with the experimental data measured previously. In generating the analytic curves, fitting parameters with 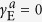, 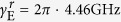, *ω*_E_ = 2*π*·9.32 GHz, 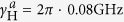, 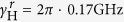 and *ω*_H_ = 2*π*·10.32 GHz have been used. Again, a good agreement between the analytic and experimental results can be identified. We remark that the theoretical explanation could be also be made by first decomposing the incident fields along the 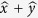 and 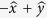 polarization directions in a one-directional manner and then evaluating the transmitted fields respectively. Comparing to such a procedure that is widely used in the literatures, our bi-directional excitation treatment emphasizes the role played by the degenerate and complementary resonances, and thus helps to illustrate the proposed general mechanism for perfect linear polarization converters.

### Design case II: a broadband linear polarization converter

Owning to the narrow magnetic resonance of the double-U structure, the polarization conversion efficiency achieved in design case I is limited within a small frequency band. To increase the conversion bandwidth, we take different approaches to realize the required perfect electric and magnetic metasurfaces with broadened responses. The results are presented here as design case II.

**Figure 4:**
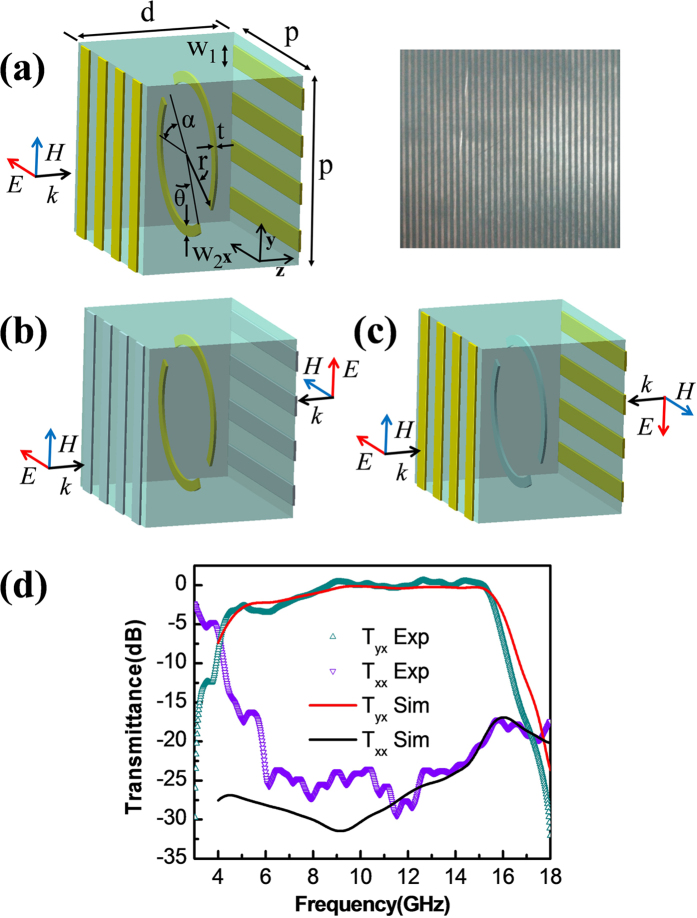
Design case II. (**a**) Schematics of the proposed device and photograph of the fabricated sample. (**b**) The proposed device under E excitation. When in resonance, the split ring is the only activated element. (**c**) The proposed device under H excitation, where the wire grating is the only activated element. (**d**) Numerically simulated and experimentally measured power transmittances for the converted and unconverted polarization components. Note that the separation between the split ring and the wire gratings, i.e. d/2, is a quarter of the resonance wavelength of the split ring structure.

Figure 4(a) sketches the unit cell of the linear polarization converter of the second case, which consists of a symmetric split ring and two wire gratings integrated in a dielectric substrate. The split ring is placed at the central plane of the substrate with openings aligned along the 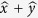 direction, and the two wire gratings are placed at the front and back ends of the substrate with grating wires aligned along the x and y directions respectively. It should be noted that such a device design falls into the category of the stacked gratings and has no fundamental difference from those studied previously by several other groups^14^,^39^,^40^. Figure 4(b) and (c) sketch the complementary resonance picture of design case II, in accordance to the situations of E and H excitations respectively. Note that because the wire gratings located at the proximal ends of the virtual sources are perpendicular to the polarizations of the incident lights, the two virtual waves can pass through them and superpose at the split ring position. For the case of E excitation, since the superposed overall electric field is polarized along the direction formed by the openings of the split ring (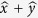), when in resonance, the split ring will act as a perfect electric conducting surface. The two incident virtual waves are thus completely reflected and the roles of the wire gratings at the distal ends are disabled. In other words, the two wire gratings are deactivated at the resonant frequency of the split ring. On the other hand, for the case of H excitation, since the superposed overall electric field is polarized perpendicular to the opening direction of the split ring (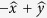), the split ring is deactivated. The two incident virtual waves thus can pass through the split ring and be reflected back by the wire gratings located at the distal ends of the virtual sources. The separation between the split ring and the distal end wire grating is set to be a quarter of the resonance wavelength of the split ring. This allows an equivalent perfect magnetic conducting surface to be created at the split ring position, provided that the operating frequency is in resonance with the split ring structure. We note that with such an arrangement, the degenerate condition for perfect electric and magnetic conducting surfaces is satisfied.

Numerical works are carried out to evaluate the performance of the device for design case II. Note that similar to design case I, here we have also assumed that the split ring and the wire gratings are made of copper and the substrate is made of F4BK. Referring to Fig. 4(a), parameters used for the numerical simulations are w_1_ = 0.8 mm, w_2_ = 0.3 mm, r = 3.7 mm, α = 45°, θ = 30°, p = 8 mm and d = 10 mm respectively. These values are determined in such a way that the electric and magnetic resonances drawn by Fig. 4(b) and (c) have degenerate resonant frequencies near 10 GHz. The solid lines in Fig. 4(d) plot the numerically obtained power transmittances of the polarization converted and unconverted components. From Fig. 4(d), it can be seen that within a broad bandwidth, the proposed device can efficiently convert the polarization of the incident wave from the x direction to the y direction. The numerical results are in good agreement with the experimental ones presented by the symbols in Fig. 4(d), which are taken from a fabricated sample shown by the right part of Fig. 4(a).

To theoretically analyze the polarization conversion process of design case II, we resort to the scattering matrix formulism. Owing to the polarization diversity, the linear polarization converter depicted in Fig. 4(a) is essentially a four-port device. The scattering matrix of such a kind four-port device reads as


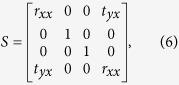


where the column indexes of 1 and 2 correspond to the x and y polarized channels incident from the left side of the metasurface respectively, and the column indexes of 3 and 4 correspond to the x and y polarized channels incident from the right side of the metasurface respectively. The scattering matrix has the following eigen-vectors


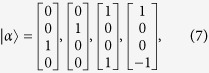


of which the two latter ones are in accordance to the E and H excitations respectively. Note that the eigen-vectors of the E and H excitations satisfy the eigen-equation of


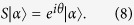


**Figure 5:**
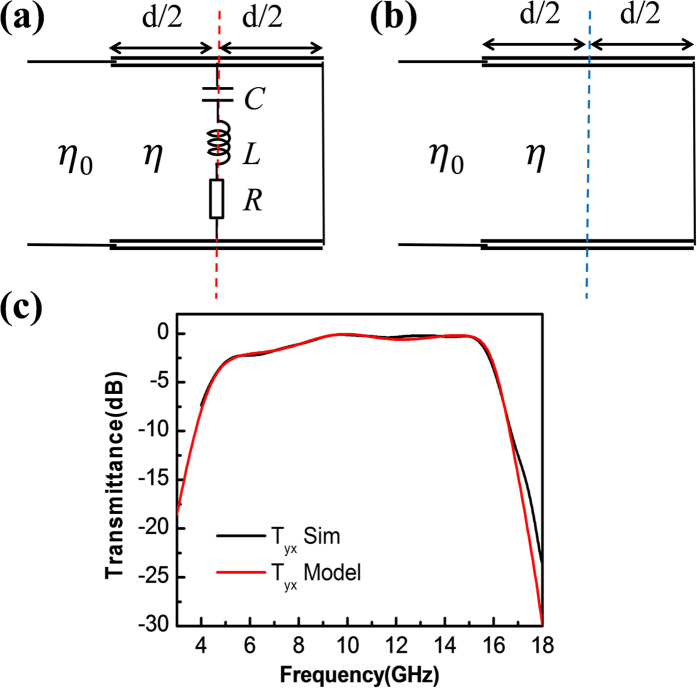
Theoretical modeling for design case II. (**a**) Transmission line model for the case of E excitation. The dashed red line indicates the position of the perfect electric conducting surface. (**b**) Transmission line model for the case of H excitation. The dashed blue line indicates the position of the perfect magnetic conducting surface. (**c**) Analytically calculated and numerically simulated power transmittances for the converted polarization component.

It indicates that the fields of the E and H excitations, when incident on the metasurface, will be totally reflected, in an analogy to the wave reflection phenomenon observed on a terminated transmission line. Inspired by such an analogy, we propose to use the transmission line model to analyze the reflection processes. Figure 5(a) depicts the transmission line model for the case of E excitation. The dielectric slab is modeled by a transmission line with a characteristic impedance of η, while the surrounding air background is modeled by a transmission line with a characteristic impedance of η_0_. The lumped circuit elements of R, L and C describe the resonance of the split ring, while the terminated short describes the wire grating placed at the distal end. Figure 5(b) depicts the transmission line model for the case of H excitation. In this case, since the split ring is deactivated, the lumped circuit elements of R, L and C are absent. Denoting the reflection coefficients for cases of E and H excitations as Γ_E_ and Γ_H_ respectively, using the standard transmission line treatment and with some manipulations, it follows that the amplitude transmittance for the polarization converted component reads as


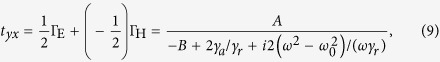


where


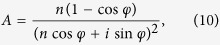



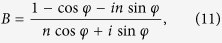


where 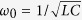, 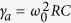, and 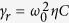 denote the resonant frequency and the absorptive and radiative losses of the split ring respectively, *n* = *η*_0_/*η* denotes the normalized refractive index of the substrate and *φ* = *ndω/c*. The analytic result calculated from Eq. (9) is plotted in Fig. 5(c) in together with the simulated data obtained previously. In generating the analytic curve, fitting parameters with *n* = 1.5, *d* = 10 mm, *γ*_*a*_ = 2*π*·0.06 GHz, *γ*_*r*_ = 2*π*·11.85 GHz and *ω*_0_ = 2*π*·9.95 GHz have been used. The good agreement between the analytic and simulated results confirms the validity of our transmission line model based analysis. We remark that the theoretical analysis could also be carried out in a more general manner using the procedure based on multiple reflection interferences^14^. However, since the roles of the complementary perfect electric and magnetic conducting surfaces are not explicitly pointed out with such a kind of treatment, the major result presented in this article, i.e. the general framework for the prefect linear polarization converter, is therefore not revealed and the fundamental connection between design cases I and II is absent.

Comparing to case I, the device realized in case II has a much larger operating bandwidth. To explain such a difference, two mechanisms are suggested in accordance to the situations of H excitation and E excitation respectively. Note that for the situation of H excitation, instead of the double-U structure [Fig. 2(c)], we use a quarter-wavelength transformed short [Fig. 5(b)] to realize the perfect magnetic conducting surface. Because the quarter-wavelength transformed short is essentially a single-mirror open cavity of which the quality factor is low (comparing to that of the double-U structure), the bandwidth of the magnetic resonance is therefore large. On the other hand, note that for the situation of E excitation, instead of the cut-wire [Fig. 2(b)], we use a split ring backed by a quarter-wavelength transformed wire grating [Fig. 5(a)] to realize the perfect electric conducting surface. Although the cut-wire and the split ring may have comparable bandwidths, however, by backing the split ring with a quarter-wavelength transformed wire grating, the bandwidth will be increased. The increase of the bandwidth can be intuitively explained by referring to Fig. 5(a) with loss effect being neglected for sake of simplicity. Note that the overall impedance at the position of the perfect electric conducting surface (marked by the dashed red line) is a parallel sum of a LC resonator and a quarter-wavelength transformed short. When the operating frequency is at the degenerate resonance, the LC resonator has a zero impedance and the quarter-wavelength transformed short has an infinite impedance. The overall impedance is thus zero and the perfect electric conducting surface condition is satisfied. When the operating frequency deviates from the degenerate resonance, the impedance of the LC resonator starts to increase from the null end to the infinity end. This renders the overall impedance also to increase away from zero and the perfect electric conducting surface condition is broken. The growing rate of the overall impedance is slow for design case II because it will be hindered by the accompanying decrease of the impedance of the quarter-wavelength transformed short from the infinity end to the null end. As a result, the response bandwidth is broadened. Note that such a mechanism has also been employed in the design of broadband linear polarization converters that operate in the reflective mode^42^.

## Discussion

We emphasize that the presented design framework for the perfect linear polarization converter bears a close resemblance to that of the perfect channel drop filter discovered previously in photonic crystal systems^43^. In essence, these two types of devices, although are functionally different, however, if viewing from a fundamental perspective, are both four-port systems, where the four ports refer to the input and output ports with x and y polarized states for the case of linear polarization converter, and to the input and output ports of the photonic crystal waveguides located at the upper and lower sides of the photonic crystal defects for the case of channel drop filter. Previous study on the photonic crystal drop filter has shown that if the involved photonic crystal defect has two degenerate resonances with odd and even symmetries, light then can perfectly tunnel from the lower side input port to the upper side output port. Taking an analogy between these two different physical systems, perfect linear polarization converter thus can be realized if the considered device possesses two perfect electric and magnetic conducting surfaces, which are in correspondence to the odd and even resonance modes of the photonic crystal defects. The above arguments illustrate that the proposed design framework for the perfect linear polarization converter is essentially a different functional application of the fundamental principle of the four-port system with appropriately arranged symmetries. It has features of being both generally applicable and physically intuitive.

Although our theoretical analysis based on Fig. 1 has predicted that a perfect linear polarization conversion is possible, however, both the numerical and the experimental investigations have shown that the conversion efficiencies are less than 100% due to the unavoidable absorptive loss and the imperfect realization of the designed devices. It should be noted that the loss related drawback, which has been encountered in many realistic design cases^4^,^13^,^27^,^30^, might be mitigated with the help of gain medium^44^ or superconducting material^45^ at the cost of increased device complexities. Another more practical route to relieve the problem has been proposed recently. It is shown that by increasing the effective inductance to capacitance ratio, i.e. L/C, the absorptive loss can be reduced substantially^46^. Note that by following this method, the reduction of the conversion loss for design case I has been confirmed numerically.

In conclusion, we have presented a framework for the design of perfect linear polarization converters. It is found that in order to achieve a 100% linear polarization conversion efficiency, the device under consideration should possess two degenerate and complementary resonances, with one being electric and the other being magnetic. We use two different design examples operating near 10 GHz to validate the proposed design framework. The obtained results show that a narrow band and a broadband linear polarization converter can be realized by following the proposed design framework, which has features of being both generally applicable and physically intuitive.

## Methods

The numerical simulations were carried out by using the frequency domain solver of a commercially available software, i.e. CST microwave studio. In our simulations, the copper structures were treated as lossy metal while the F4BK substrate was treated as normal.

The experimental measurements were performed in an anechoic chamber. A pair of linearly polarized broadband horn antennas were placed in front of and behind a transmission window of a microwave absorbing screen, acting as the transmitter and receiver of the measurement system. Fabricated device samples were mounted on the transmission window of the microwave absorbing screen. A vector network analyzer (Agilent N5244) was utilized to measure the polarization related transmittances of the fabricated devices, with the free space case served as the normalization reference. Because the transmitting and receiving antennas are not impedance matched to the free space, residue reflections occur at their coupling planes. The residue reflections from the antennas and also from the samples form a Fabry-Perot alike effect and manifest themself as ripples in the measured transmittance curves. Note that the frequency of the measured ripple is in good agreement with that estimated from the separations between the antennas and the samples.

## Additional Information

**How to cite this article**: Zhou, G. *et al*. Designing perfect linear polarization converters using perfect electric and magnetic conducting surfaces. *Sci. Rep.* **6**, 38925; doi: 10.1038/srep38925 (2016).

**Publisher's note:** Springer Nature remains neutral with regard to jurisdictional claims in published maps and institutional affiliations.
